# Molecular Characterization of Muellerian Tumors of the Urinary Tract

**DOI:** 10.3390/genes12060880

**Published:** 2021-06-07

**Authors:** Nadina Ortiz-Brüchle, Sophie Wucherpfennig, Michael Rose, Stefan Garczyk, Simone Bertz, Arndt Hartmann, Henning Reis, Tibor Szarvas, András Kiss, Felix Bremmer, Reinhard Golz, Ruth Knüchel, Nadine T. Gaisa

**Affiliations:** 1 Institute of Pathology, RWTH Aachen University, 52074 Aachen, Germany; nortiz-bruechle@ukaachen.de (N.O.-B.); swucherpfenn@ukaachen.de (S.W.); mrose@ukaachen.de (M.R.); sgarczyk@ukaachen.de (S.G.); rknuechel-clarke@ukaachen.de (R.K.); 2 Center for Integrated Oncology Aachen Bonn Cologne Duesseldorf (CIO ABCD), 52074 Aachen, Germany; 3 Institute of Pathology, University Hospital Erlangen, Friedrich-Alexander-Universität Erlangen-Nürnberg, 91054 Erlangen, Germany; simone.bertz@uk-erlangen.de (S.B.); arndt.hartmann@uk-erlangen.de (A.H.); 4 Institute of Pathology, West German Cancer Center, University of Duisburg-Essen, University Hospital Essen, 45147 Essen, Germany; Henning.Reis@uk-essen.de; 5 West German Cancer Center, Department of Urology, University of Duisburg-Essen, University Hospital Essen, 45147 Essen, Germany; sztibusz@gmail.com; 6 Department of Urology, Semmelweis University, 1085 Budapest, Hungary; 7 2nd Department of Pathology, Semmelweis University, 1085 Budapest, Hungary; kiss.andras@med.semmelweis-univ.hu; 8 Institute of Pathology, University Medical Center, University of Göttingen, 37075 Göttingen, Germany; felix.bremmer@med.uni-goettingen.de; 9 Institute of Pathology, HELIOS Clinic Wuppertal, 37075 Wuppertal, Germany; reinhard.golz@helios-gesundheit.de

**Keywords:** Muellerian tumors, clear cell adenocarcinoma, endometrioid adenocarcinoma, ARID1A, TERT

## Abstract

In the 2016 WHO classification of genitourinary tumors Muellerian tumors of the urinary tract (MTUT) comprise clear cell adenocarcinomas and endometrioid carcinomas. Since these rare tumors remained understudied, we aimed to characterize their molecular background by performing DNA- and RNA-based targeted panel sequencing. All tumors (*n* = 11) presented single nucleotide alterations (SNVs), with *ARID1A* mutations being the most prevalent (5/11, 45%). Besides frequent *ARID1A* mutations, loss of ARID1A protein is not a suitable marker since protein expression is (partly) preserved also in mutated cases. Copy number alterations (CNVs) were found in 64% of cases (7/11), exclusively gene amplifications. Interestingly, a functionally relevant *RSPO2* gene fusion/microdeletion was discovered in the endometrioid adenocarcinoma case. Comparing our findings with mutational profiles of other tumor entities, absence of *TERT* promoter mutations argues for a non-urothelial origin. No similarities were also found between MTUT and kidney cancers while parallels were observed for specific SNVs with endometrial carcinomas. In conclusion, immunohistochemical PAX8-positivity and lack of *TERT* promoter mutations could serve as key diagnostic features in difficult cases. Thus, understanding the molecular background of these tumors helps to refine treatment options and offers the possibility of targeted therapies in cases where needed.

## 1. Introduction

Muellerian-type tumors of the urinary tract (MTUT) were implemented in the 2016 WHO classification as an entity comprising clear cell adenocarcinomas (CCAs) and endometrioid adenocarcinomas (EAs) of the bladder/urinary tract [1]. The histologically clear cell tumors were initially described as mesonephric adenocarcinomas by Konnak in 1973 [2] and re-named as clear cell adenocarcinomas in 1985 by Young and Scully [3]. They are rare malignancies and occur preferably in women (female-to-male ratio 2:1) or in case of endometrioid adenocarcinomas exclusively in women [1]. However, recently, a series of *n* = 15 CCAs in men was also reported [4]. Most of the CCAs are located in the bladder neck and trigone, less frequently in the lower posterior bladder wall, whereas EAs are located in the trigone and the posterior wall, where endometriosis is most common [1]. Due to lack of information, prognosis of EAs is not assessable [1]. Prognosis of CCAs depends on tumor growth/stage (exophytic tumors are more favorable) and aggressive treatment [1], since theses tumors respond poorly to chemotherapy or radiotherapy and show the best long term survival after radical cystectomy [5].

Diagnostically striking features are hobnail-like tubulocystic, papillary, or diffusely arranged cells with either clear glycogen-rich or eosinophilic cytoplasm (clear cell adenocarcinomas) or typically endometrioid features with squamoid nests (endometrioid adenocarcinomas) [1]. Secondary bladder infiltration from tumors of neighbor organs must be excluded. The most helpful immunohistochemical markers are PAX2 or PAX8, which are positive in all cases [6], but keratin7, EMA, HNF1ß, and CA125 are usually positive as well [1]. The immunohistochemical profile of endometrioid adenocarcinomas is similar to the female genital counterparts, including hormone receptor expression [1].

Due to the low incidence rates, the biological background and implications for clinical management are not yet well understood, and first reports are now available [7]. In the latest study of Lin et al., they analyzed four CCAs by targeted panel sequencing and identified BK virus-mediated oncogenesis (one of four cases) and PI3K/AKT/mTOR pathway activation (three of four cases) as underlying tumorigenic events, waiting for further validation in lager cohorts [7]. Due to these findings, we hypothesized that clear cell adenocarcinomas might be closer related to genital clear cells and nephrogenic cells than urothelial cells. Therefore, in the presented work, we collected Muellerian-type tumors and analyzed their molecular alterations on SNV, CNV, and gene fusion level and compared it with available TCGA data from bladder, endometrioid, and clear cell kidney cancers in order to understand tumorigenesis and identify new treatment options.

## 2. Materials and Methods

### 2.1. Study Cohort

In total, 11 MTUTs were collected from various collaborating Institutes of Pathology. All cases were re-evaluated histomorphologically on H&E sections by an expert genitourinary pathologist (NTG). Clinical and morphological characteristics of the cohort are shown in Table 1. The study was a retrospective, anonymous study on archival tissue samples and was approved by the local ethics committee (RWTH Aachen University EK 415/19).

### 2.2. Immunohistochemical confirmation of Diagnosis

All samples underwent immunohistochemical confirmation of diagnosis. Whole representative tissue slides were stained with a limited marker panel (keratin7, PAX8, and GATA3) and only strongly PAX8-positive tumors (>50% strong nuclear positivity) were accepted. Additional staining for ARID1A was performed in cases with sufficient material after mutational analysis. Deparaffinization and antigen retrieval was performed with DAKO PT-Link heat induced antigen retrieval with low pH (pH 6) Target Retrieval Solution (DAKO, Hamburg, Germany). For stainings, the slides were incubated for 20–30 min (Ready-to-Use) at room temperature with anti-Cytokeratin 7 (clone OV-TL 12/30, Ready-to-Use, Flex+M; Agilent Technologies/DAKO, Hamburg, Germany), anti-PAX8 (clone MRQ-50, dilution 1:100, Flex+M; Cell marque/Merck KGaA, Darmstadt, Germany), anti-ERBB2/Her2 (polyclonal, dilution 1:300, DAKO), and anti-GATA3 (clone L50-823, dilution 1:250, Flex+M; Biocare Medical, Pacheco, California, USA/Zytomed Systems GmbH, Berlin, Germany) in a DAKO Autostainer (DAKO). For anti-ARID1A (1:250, D2A8U, Cell Signaling), estrogen receptor (clone 1D5, dilution 1:60, DAKO), and progesterone receptor (clone PgR636, DAKO, ready-to-use), high pH (pH9) Target Retrieval Solution (DAKO) was used and incubated for 60 min at room temperature. For visualization, the appropriate linker molecules EnVision^TM^FLEX+ (mouse/rabbit), the EnVision FLEX/HRP detection system, and a counterstaining with EnVision FLEX Hematoxylin were applied.

### 2.3. DNA and RNA Isolation

Manual microdissection of tumor tissue for target values of 70–80% tumor cell content (one outlier sample with 40% tumor cells) was conducted after annotation of the regions-of-interest by the expert genitourinary pathologist (NTG) on H&E slides. DNA isolation was performed using the Maxwell^®^ DNA preparation kit (Maxwell^®^ 16 System, Promega, Mannheim, Germany) according to the manufacturer’s instructions. RNA was isolated similarly by using Maxwell^®^ 16 LEV RNA Purification FFPE Kit (Promega) according to the manufacturer’s instructions.

### 2.4. Targeted Next Generation Sequencing

For NGS, an amplicon panel (AmpliSeq for Illumina Comprehensive Panel V3, FA: Illumina, San Diego, CA, USA) covering 161 genes (DNA: 134 genes, RNA: 51 genes) known to be frequently altered in various cancers was used. DNA and RNA libraries were prepared according to the manufacturer’s protocols, and sequencing was conducted on a NextSeq500 sequencer or MiSeq sequencer (Illumina; reference genome GRCh37/hg19) for *n* = 11 samples. Tumor cellularity ranged from 40–80% in all cases. For DNA sequencing, all samples exceeded 20 million, and for RNA, 650,000 total passed filter reads/sample. Bam-file generation was performed with the DNA amplicon module (DNA Amplicon Workflow, Version 3.7.13-O00399LRM-DAv1, Illumina). Alignment and variant calling were done using Sequence Pilot Software version 4.4.0 Build 509 (SeqNext module; JSI Medical Systems, Ettenheim, Germany).

Variants with an allele frequency above 10% and a coverage of at least 200× were further evaluated if not already classified as known artifacts for the panel. Further filtering was carried out as follows: missense variants with an allele frequency >2% in healthy controls (according to 1000 Genomes (http://www.internationalgenome.org, accessed on 15 April 2021) or dbSNP v154 (https://www.ncbi.nlm.nih.gov/snp, accessed on 15 April 2021)), non-splicing–relevant synonymous, and intronic variants not affecting the canonical splice-site as well as variants of the untranslated regions (UTRs) were considered benign. Furthermore, missense variants classified as benign or likely benign in the ClinVar database (https://www.ncbi.nlm.nih.gov/clinvar, accessed on 15 April 2021, 2021) were excluded. The OncoKB database (https://www.oncokb.org/, accessed on 15 April 2021) was used for further pathogenicity assessment and for a classification of genes into tumor suppressor genes and oncogenes. For copy number variation (CNV) analysis, an in-house algorithm was used (ACopy). It was validated using three NGS panels (>150 samples) and is based on a model for the efficiency of PCR exponential growth of single amplicons in all measured samples [8]. For visualization of variants, oncoprints were created with OncoPrinter on http://cbioportal.org [9,10]. Fusion gene analysis was carried out with the RNA amplicon module (RNA Amplicon Workflow, Version 0.17.0.595+RAv1, Illumina).

### 2.5. Fluorescence In Situ Hybridization Analysis (FISH)

ZytoLight Dual Color Probes SPEC *MET/CEN 7* and SPEC *Her2/CEN 17* (Zytovision, Bremerhaven, Germany) were hybridized onto 3 µm tissue sections according to the manufacturer’s protocols. Slides were evaluated with a Zeiss Axiovert 135 fluorescence microscope (Carl Zeiss, Oberkochen, Germany), and Diskus Software (Büro Hilgers, Königswinter, Germany) was used to capture images from different channels/filters (AHF ZyGreen F36-720, AHF ZyOrange F36-740, AHF DAPI, AHF F56-700). The numbers of locus signals and centromere signals were counted in 20–60 nuclei of tumor cells at high magnification (×1000), and a locus/centromere ratio as well as a locus/cells ration were calculated.

### 2.6. Comparison with Publicly Available TCGA Data of Bladder, Endometrial, and Kidney Cancer

Publicly available datasets from The Cancer Genome Atlas (TCGA) were used to compare SNV and CNV data of the presented study with further cancer entities, i.e., bladder [11], endometrial [12], and kidney [13] cancer. SNVs, CNVs, and clinico-pathological data were obtained using the cBioPortal platform (https://www.cbioportal.org/, [9,10]) filtering for the identified genetic alterations of this study.

## 3. Results

### 3.1. Histomorphological and Immunohistochemical Diagnostic Evaluation

In total, 10 cases showed typical clear cell morphology with a predominantly papillary architecture in 50% (5/10) of cases, followed by 30% (3/10) tubulo-cystic pattern, and only 20% (2/10) of cases exhibited a predominant solid clear cell tumor mass.

**Figure 1:**
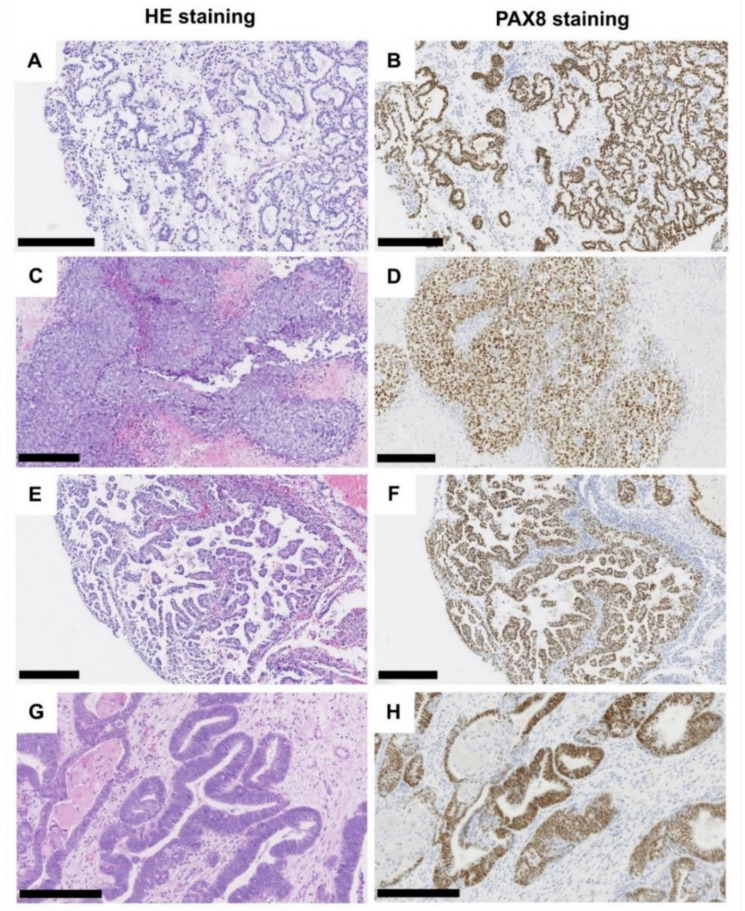
Histological characteristics of tumors of the Muellerian type. Clear cell adenocarcinoma, tubule-cystic growth pattern (**A**), HE, (**B**) PAX8 immunohistochemistry. Clear cell adenocarcinoma, solid growth pattern (**C**), HE, (**D**) PAX8 immunohistochemistry. Clear cell adenocarcinoma, (pseudo-/micro-) papillary growth pattern (**E**), HE, (**F**) PAX8 immunohistochemistry. Endometrioid adenocarcinoma (**G**), HE, (**H**) PAX8 immunohistochemistry. Black scale bar: 250 µM.

One case showed the typical endometrioid histology with a glandular pattern and more or less distinct squamous areas. All cases presented with invasive growth and revealed a strong nuclear positivity for PAX8, medium to strong cytoplasmatic and membranous positivity for keratin7, and only weak to absent staining for GATA3 (see Table 1). Figure 1 illustrates the different histomorphological phenotypes of MTUT.

### 3.2. Mutational Analysis

**Figure 2:**
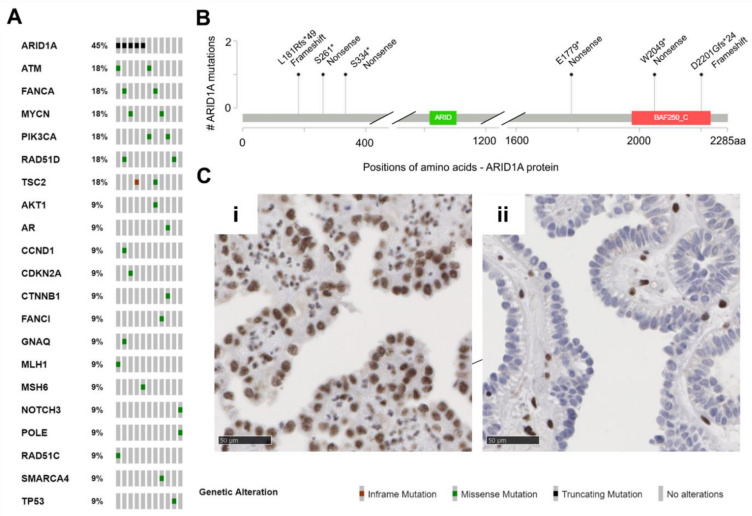
Muellerian tumors of the urinary tract (MTUT) are characterized by frequent mutations in *ARID1A*. (**A**) Oncoprint image illustrates single nucleotide variations (SNVs) including in-frame mutations, missense mutations, truncating mutations. (**B**) Positions and type of *ARID1A* mutations identified in MTUT. (**C**) ARID1A protein expression/loss in exemplary MTUT tissues. (**i**) MTUT tissue with *ARID1A* wild type shows strong nuclear staining of ARID1A protein. (**ii**) ARID1A protein loss in MTUT tissue with p.Ser334* *ARID1A* mutation. Black scale bar: 50 µM.

DNA NGS panel analysis was successfully performed for all 11 samples. Region-of-interest coverage was at least 91% (up to 98%) at 500× coverage (average 95%). A total of 32 variants of pathogenic or at least uncertain significance emerged after filtering (Figure 2B; Table S1). The identified mutations were 25 missense mutations (78%), six truncating mutations (19%), and one in-frame deletion (3%). A total of 22 variants were detected in genes classified as tumor suppressors according to the OncoKB database (69%), whereas nine variants were found in corresponding oncogenes (28%). For one variant of the *NOTCH3* gene, both functions were described according to OncoKB (3%).

No identical recurrent mutations were detected. Except for one *ARID1A* mutated case, in none of the tumor suppressor genes were two mutations of the same gene detected (patient MT-10). The most frequently mutated gene (5/11 patients, 45%, 6/32 variants 19%) in our cohort was *ARID1A* (Figure 2B). Interestingly, all six detected variants were clearly truncating mutations, which resulted in loss of protein in immunohistochemical analysis in only one patient (MT-6: p.Ser334*, Figure 2Cii). Two further *ARID1A* mutations led to a partial protein loss (MT-4: p.Ser261*, MT-5: p.Asp2201Glyfs*24), and the residual mutations (MT-7: p.Leu181Argfs*49) showed retained ARID1A protein expression or immunohistochemistry was not possible (MT-10: p.Glu1779*, p.Trp2049*). Further, mutations of *ATM*, *FANCA*, *MYCN*, *PIK3CA*, *RAD51D*, and *TSC2* genes were observed two times each, while single changes in other genes were detected only once (*AKT1*, *AR*, *CCND1*, *CDKN2A*, *CTNNB1*, *FANCI*, *GNAQ*, *MLH1*, *MSH6*, *NOTCH3*, *POLE*, *RAD51C*, *SMARCA4*, *TP53*).

To reliably exclude *TERT* promoter mutations, in particular, the known hotspot mutations c.-146C>T (“C250T”) c.-124C>T (“C228T”), we performed a separate coverage analysis with respect to *TERT* [14]. For all samples, a minimum coverage of 100× in the range of the two mentioned mutations was achieved (90.9% of cases >300×, 63.6% of cases >500×). A *TERT* promoter mutation could not be identified in any of the 11 patients.

### 3.3. Copy Number Analysis

All 11 analyzed samples passed our internal CNV quality assessment (quality scores ranged from 97%–100%, mean value: 99%). In total, we identified 13 copy number alterations (only amplifications) in seven samples of our cohort. Nine of the 13 detected CNVs affected oncogenes (69%), whereas four of the detected amplifications involved tumor suppressor genes (31%).

**Figure 3:**
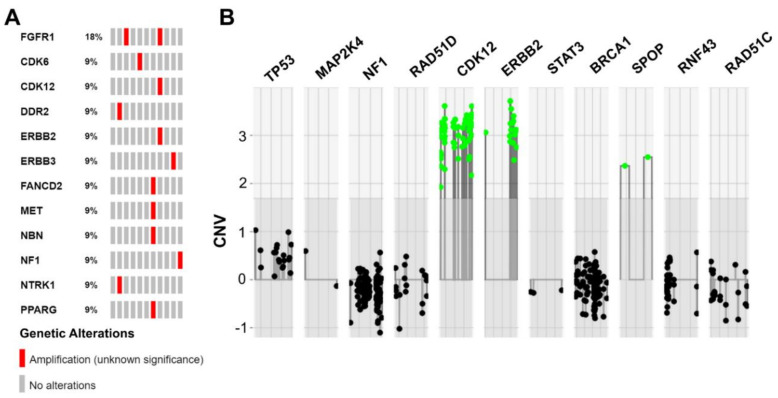
Summarized copy number variations (CNVs) identified in MTUT. (**A**) Oncoprint image illustrates detected CNVs. (**B**) Illustration extracted from the ACopy tool [8] showing the co-occurring amplification of *ERBB2* and *CDK12* in patient MT-8 (x-axis: 0 corresponds to two copies).

In one patient, four CNVs were detected. However, two of the CNVs were located on chromosome 3p25.2–3p25.3 in spatial proximity to each other, therefore, a larger chromosomal event seems to be likely in this case (patient MT-7: 3p25.2–3p25.3: 1× *FANCD2*, 1× *PPARG*; additionally: 1× *MET*, 1× *NBN*). Another patient harbored three CNVs, with two of the affected genes chromosomally adjacent (patient MT-8: 1× ERBB2, 1× *CDK12*: chromosome 17q12; also: 1× *FGFR1*). Additionally, in the last case with multiple CNVs, amplifications of two genes in spatial proximity were detected (patient MT-2: 1× *NTRK1*, 1× *DDR1*; chromosome 1q23.1–1q23.3). The remaining four cases showed one amplification event each (1× *FGFR1*, 1× *CDK6*, 1× *ERBB3*, 1× *NF1*). Therapeutically relevant amplifications were further evaluated by FISH. *MET*-FISH revealed a polysomy with four to six copies of chromosome 7 (ratio *MET* signals (132)/CEN 7 (104) = 1.27 and ratio *MET* signals (132)/number of cells (20) = 6.6). *ERBB2*-FISH proved a true amplification of *ERBB2* (ratio *ERBB2* signals (324)/CEN 17 (132) = 2.45) with one to three copies of chromosome 17 and immunohistochemically strong baso-lateral Her2neu staining. A summary of the CNV data is shown in Figure 3, Table S2.

### 3.4. Fusion Gene Analysis

**Figure 4:**
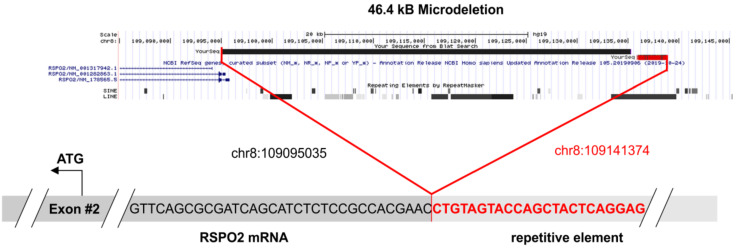
The 46.4 kB microdeletion of patient MT-2. In the upper panel, the deleted sequence is demonstrated. The chromosomal region was extracted from the UCSC genome browser (https://genome.ucsc.edu/, accessed on 15 April 2021) and the exact breakpoints are listed. The lower panel shows the fusion of the intronic repetitive element to the first (non-coding) base of exon 2. The sequence data were extracted from the original fastq file and were aligned to the reference genome GRCh37/hg19.

Analysis of fusion genes on RNA level was successfully performed for all 11 patients. At least 230,000 single RNA reads were obtained per case (range: 235,710–4,770,440, mean value: 1,127,386 reads). A total of 10 of the cases did not show gene fusions, however, in one patient with the endometrioid adenocarcinoma (MT-2), an *RSPO2* rearrangement was detected, which was already described as functionally relevant in hepatocellular adenoma (*n* = 3/17, [15]). The fusion connects an intergenic short interspersed nuclear element (SINE) upstream of *RSPO2* (chr8:109141374) to exon 2 of the *RSPO2* gene (chr8:109095035). Ultimately, 16,750 supporting reads were obtained. Analysis of the fastq files confirmed the breakpoints of the 46.4 kb microdeletion on chromosome 8q23.1 (Figure 4, Table S3).

### 3.5. Comparison with Publicly Available TCGA Data of Bladder, Endometrial, and Kidney Cancer

**Figure 5:**
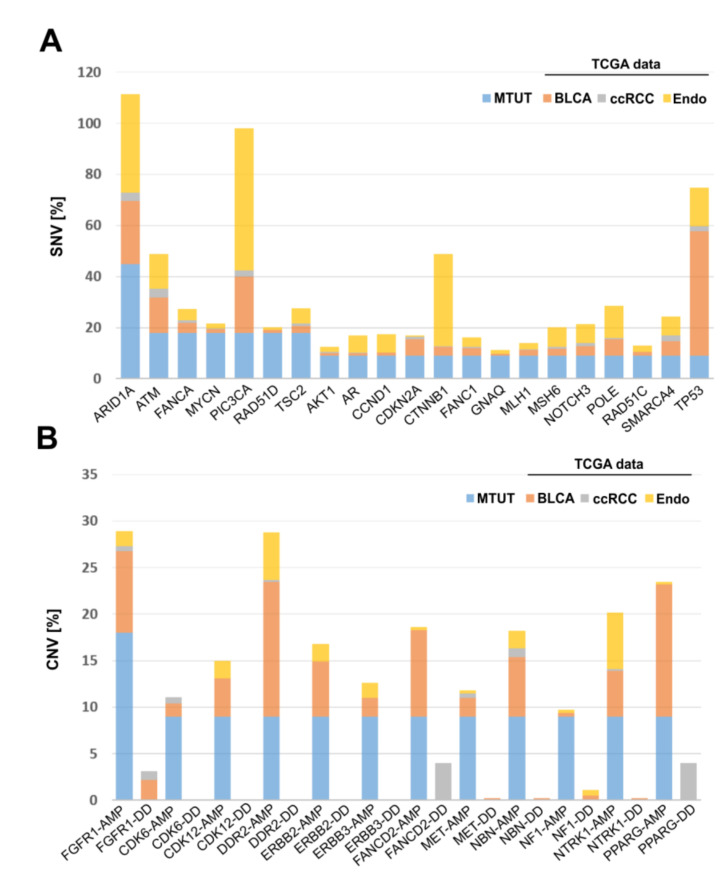
Comparison of genetic alterations found in MTUT with TCGA data sets of bladder, endometrial, and clear cell kidney cancers. (**A**) SNVs are shown. (**B**) CNVs are illustrated. AMP: amplification; DD: deep deletion, BLCA: bladder cancer, ccRCC: clear cell renal cell carcinoma.

Finally, the identified SNV and CNV alterations in Muellerian-type tumors of the urinary tract were compared with cancers of other origin, i.e., with bladder (BLCA, *n* = 412), endometrial (*n* = 205), and clear cell kidney (ccRCC, *n* = 426) cancer of the TCGA platform. SNVs are illustrated in Figure 5A and revealed the lowest similarity between MTUT and clear cell kidney cancers, showing overall very low mutational frequency of analyzed genes. Contrary to that, we identified distinct gene alterations such as *ATM* and *POLE* present in MTUT with comparable frequencies in both ±3%, i.e., bladder (*ATM*: 13.8, *POLE*: 6.6) and endometrial cancers (*ATM*: 13.7, *POLE*: 12.7), while genes such as *PIC3CA* or *TP53* showed only parallels with one of the two entities. Interestingly, we also observed gene alterations which seemed to be more specific for MTUT such as *MYCN* and *RAD51D.*

Focusing on CNVs (Figure 5B), ccRCC exhibited amplifications of analyzed genes only in rare cases, whereas deep deletions were much more frequent for *PPARG* and *FANCD2*. No similarities were observed when comparing MTUT and endometrial cancers, showing only an increased amplified status for *DDR2*. In turn, CNV data revealed parallels between bladder cancers and MTUT for most of analyzed genes, such as *FGFR1,* according to both frequency and type of CNV, i.e., amplifications were mainly present, whereas deep deletions occurred rarely.

## 4. Discussion

We collected—to our knowledge—the largest cohort of MTUTs thus far, a rare and only recently added/renamed tumor group of urinary tract in the 2016 WHO classification. The presented study is the most comprehensive molecular analysis on MTUTs with focus on molecular changes in order to understand tumorigenesis and including therapeutically relevant targets thus far.

DNA panel sequencing revealed 32 potentially pathogenic variants in our MTUT cohort distributed among all analyzed patients and 13 CNVs (amplifications only) affecting only seven cases of our cohort. Comparing the mutational spectrum of Muellerian tumors with external data of bladder, kidney, and endometrial cancers from the TCGA project revealed no molecular similarity with clear cell kidney cancers. However, this does not fully argue against a possible origin from nephrogenic adenoma, since neighboring nephrogenic adenoma and clear cell adenocarcinomas were described and previous reports showed similar chromosome 9 and 17 loss of heterozygosity (LOH) in CCAs and identical changes at chromosomes 1, 4, and 8 in both lesions [16]. There were variable similarities for single genes with endometrial and bladder cancer, but the absence of *TERT* promoter mutations, the most frequent mutational alterations identified in urothelial carcinomas [17], argues for a non-urothelial origin. Since *TERT* promoter mutations, genetic alterations that drive TERT expression, are widespread throughout different stages and grades of the disease, it is hypothesized that they are the basis of urothelial cancer initiation [18]. A recent pan-cancer study which included sequencing of *n* = 796 urothelial cancers confirmed high frequencies (>70%) of *TERT* promoter mutations in urothelial carcinomas of the urinary bladder and the urethra and a slightly reduced prevalence in upper tract urothelial cancers (53%) [18]. Contrary to that, renal cell carcinomas showed only low numbers of *TERT* mutations (~10%), whereas more than 1000 endometrial carcinomas are rarely characterized by *TERT* alterations (<5%) [19]. The reason for the strong differences of mutational frequencies of *TERT* between cancer entities is not well understood. Chiba and colleagues recently revealed that *TERT* promoter mutations did not prevent telomere shortening but could keep the shortest telomeres intact, while advanced telomeres are critically short, leading to genomic instability [20]. These data might support the hypothesis that *TERT* mutations are not the only drivers to initiate an oncogenic process, explaining also their presence in premalignant lesions and non-TERT expressing tumors [21]. However, our findings may help in diagnostically very difficult MTUT cases, since a wild type *TERT* promoter mutation analysis could serve as an additional tool to guide pathologists as well as clinicians in diagnosis and management. Comparing the data with our previous characterization of genetic alterations of intestinal type adenocarcinomas of the bladder, it parallels with clear cell adenocarcinomas, since genes such as *ARID1A* (intestinal type: 30.6%), *CTNNB1* (intestinal type: 11.1%), and *MSH6* (intestinal type: 8.3%) showed similar mutational frequencies between both carcinomas [22]. In contrast, *TP53* mutations seem to be much more predominant in intestinal type adenocarcinomas of the bladder (31 out of 36) than in clear cell adenocarcinomas of the bladder/urethra (1 out of 11).

As is implicated by the term “Muellerian-type” tumors and the female preponderance, they are currently thought to arise from preexisting Muellerian precursors within the urinary bladder. Hormone receptor positivity in our endometrioid adenocarcinoma arising from clinically reported endometriosis supports this notion, however, all of our CCA cases were negative for hormone receptors. Furthermore, in some cases, CCAs are also associated with urothelial carcinoma, urothelial carcinoma in situ, and adenocarcinoma, thus arguing also for a type of divergent differentiation [1]. Our molecular data are currently more supportive of a common Muellerian origin (Ductus paramesonephricus, giving rise to tube, uterus, and upper vagina), however, sequencing of larger genomic areas and extended sample cohorts is necessary.

Most detected variants affected the Fanconi anemia/DNA repair pathway (34%, *ATM, FANCA*, *FANCI*, *MLH1*, *MSH6*, *POLE*, *RAD51C*, *RAD51D*) followed by mutations altering the SWI/SNF (SWItch/sucrose non-fermentable) complex (22%, *ARID1A* and *SMARCA4*). In line with previous limited data, we also found several alterations in the phosphoinositide 3-kinase (PI3K) pathway (16%, *AKT1*, *PIK3CA*, *TSC1*, *TSC2*) [7]. Less frequently, alterations affected the cell cycle regulation (6%, *CCND1*, *CDKN2A*), the Myc pathway (6%, *MYCN*), and others such as p53 signaling (3%, *TP53*), G protein-coupled receptor signaling (3%, *GNAQ*), notch signaling (3%, *NOTCH3*), WNT signaling (3%, *CTNNB1*), and androgen receptor signaling (3%, *AR*). Interestingly, the mitogen-activated protein kinases/extracellular signal-regulated kinases (MAPK/ERK) pathway was affected in over 50% of detected amplifications (54%, *ERBB2*, *ERBB3*, *FGFR1*, *NF1*, *NTRK1*, *MET*), although no point mutation was found in the MAPK/ERK pathway genes. Other involved pathways were again cell cycle signaling (15%, *CDK6*, *CDK12*), Fanconi anemia/DNA repair (15%, *FANCD2*, *NBN*), and additionally peroxisome proliferator-activated receptor (PPAR) signaling (8%, *PPARG*) as well as discoid in domain receptor 2 signaling (8%, *DDR2*).

For most of the signaling pathways mentioned, disruption is known to have a strong driving effect on tumor development. Therefore, as with PI3K or DNA repair signaling pathways, they represent desirable and important targets for targeted therapies in advanced stage metastasized patients [23,24]. In general, an accumulation of alterations in DNA repair genes in a broader sense is striking in our cohort. Alterations affecting DNA repair are the focus of intense current research and clinical studies of several cancer types. For example, PARP inhibitors were FDA approved in prostate cancer with BRCA1/2 alterations and other homologous recombinational repair (HRR) genes such as ATM [25].

For more than 80% (26/32) of the detected alterations in our MTUT cohort, a search for clinical trials at https://www.clinicaltrials.gov/ (accessed on 15 April 2021) revealed at least one phase 1 and/or 2 trial of a targeted drug, including, e.g., ATR inhibitors (NCT02278250), CDK4/6 inhibitors (NCT03297606), and PARP inhibitors (NCT04171700), currently ongoing in solid tumors. Additionally, there are even FDA approved drugs available for some of the discovered alterations, such as trastuzumab for *ERRB2* amplifications or alpelisib for *PIK3CA* mutations in breast cancer [23,26]. Although none of the potential targeted therapies are currently used in MTUT patients, our data indicate that (advanced stage) patients may benefit from genetic testing in the future in order to uncover the spectrum of potentially druggable alterations. For clinical purposes, these alterations should be discussed in an interdisciplinary molecular tumor board addressing the synopsis of molecular and histo-pathological data as well as the clinical situation of the patient and providing an individual therapeutic suggestion.

*ARID1A* was the most commonly altered gene in our MTUT cohort (45%). Interestingly, all detected variants were truncating and thus clearly inactivating. One sample harbored two mutations in *ARID1A*, therefore, either a biallelic loss or intratumoral heterogeneity can be assumed. Unfortunately, for this case (MT-10), no tissue for immunohistochemical confirmation was available. However, only for one of the five cases analyzed by immunohistochemistry, a complete loss of expression could be confirmed (MT-6, although only harboring one mutation). For the other mutated cases, a partial expression (MT-4, MT-6) or a regular expression (MT-7) were observed. Nevertheless, our results are consistent with observations from other studies. Wu and Roberts summarize that, for example, in one study, in only 30% of ovarian clear cell carcinomas with *ARID1A* mutations were both alleles affected by mutations. A total of 73% of tumors with heterozygous *ARID1A* mutations lacked expression on protein level analyzed by immunohistochemistry. Discussed causative mechanisms include loss of heterozygosity, mutations in non-coding regions of *ARID1A,* or post-transcriptional and/or post-translational mechanisms [27]. In turn, 27% of *ARID1A* heterozygous ovarian clear cell carcinomas exhibited detectable protein expression. A systematic loss-of-heterozygosity (LOH) analysis was not performed due to methodological reasons, nevertheless, in one case (MT-4) with only partial ARID1A expression in immunohistochemistry, we observed a synonymous assumed—although rare—germline single nucleotide polymorphism (c.5400T>C, p.Asn1800=; rs544579117). Supposing heterozygosity, one would expect an allele frequency of approximately 50%, but in this case, a shifted allele frequency of 74% was observed. This could be an indication of a partial (copy number neutral) LOH in some tumor cells and could therefore be an explanation for the observed partial loss of expression in immunohistochemistry. Overall, the observations that *ARID1A* can harbor heterozygous mutations but still be expressed from the unaffected allele raise the question of the extent to which reduced expression of ARID1A might have a haploinsufficiency effect and thus contribute to carcinogenesis [28].

ARID1A is part of the SWI/SNF complex, a subfamily of ATP-dependent chromatin remodeling complexes, and is mutated in about 20% of all cancers across entities [29]. Inactivation of the SWI/SNF complex is proposed to be a promising biomarker for several targeted drugs. It was observed that ARID1A loss of function sensitizes cells to PARP inhibitors [30]. Other data suggest that immunotherapy might be a rational therapy for ARID1A-deficient carcinomas [31]. Currently, several studies are ongoing involving ATR inhibitors, with first promising results regarding measurable response, at least in single patients [32]. Therefore, ARID1A emerges as an attractive candidate for targeted therapies.

## Figures and Tables

**Table 1:** Clinical and morphological characteristics.

	MTUT	CCACA	EACA
	*n* (%)	*n* (%)	*n* (%)
**Total number of cases**	11 (100)	10 (91)	1 (9)
**Gender**			
male	2 (18)	2 (100)	0 (0)
female	9 (82)	8 (89)	1 (11)
**Localization**			
trigone	3 (27)	2 (67)	1 (33)
bladder neck	1 (9)	1 (100)	0 (0)
urethra/periurethral	6 (55)	6 (100)	0 (0)
not available	1 (9)	1 (100)	0 (0)
**Dominant histological growth pattern**			
tubulo-cystic	3 (27)	3 (100)	0 (0)
(micro-)papillary	5 (45)	5 (100)	0 (0)
solid	2 (18)	2 (100)	0 (0)
not applicable/special	1 (9)	0 (0)	1 (100)
Stage distribution *			
pT1	3 (27)	3 (100)	0 (0)
pT2	2 (18)	1 (50)	1 (50)
pT3	1 (9)	1 (100)	0 (0)
n.a.	5 (46)	5 (100)	0 (0)
Grade distribution (WHO 1973) *			
G2	2 (18)	1 (50)	1 (50)
G3	2 (18)	2 (100)	0 (0)
not available	7 (64)	7 (100)	0 (0)
**Grade distribution (WHO 2016) ^#^**			
high-grade	11 (100)	10 (91)	1 (9)
**Immunohistochemistry**			
keratin7 positivity	6/9 (67)	5 (83)	1 (17)
keratin20 positivity	2/6 (33)	2 (100)	0 (0)
GATA3 positivity	3/10 (30)	2 (67)	1 (33)
PAX8 positivity	11/11 (100)	10 (90)	1 (10)
ARID1A positivity	6/7 (86)	5 (83)	1 (17)
Estrogen receptor positivity	1/8 (13)	0 (0)	1 (100)
Progesterone receptor positivity	1/8 (13)	0 (0)	1 (100)

MTUT: Muellerian tumor of the urinary tract, CCACA: clear cell adenocarcinoma, EACA: endometrioid adenocarcinoma, WHO: World Health Organization, * according to the original diagnostic files, ^#^ according to the review by NTG.

## Supplementary Materials

The following are available online at https://www.mdpi.com/article/10.3390/genes12060880/s1, The datasets supporting the conclusions of this article are included within the article and its additional files: Table S1: Mutations detected by NGS in the Muellerian tumors of the urinary tract cohort. Table S2: Copy number variations (CNVs) detected by NGS in the Muellerian tumors of the urinary tract cohort. Table S3: Details on RSPO2-Fusion/Microdeletion.
